# Supplementary material for: A super liquid-repellent hierarchical porous membrane for enhanced membrane distillation
Source: Nat Commun. 2023 Oct 28;14:6886. doi: 10.1038/s41467-023-42204-7 (PMC10613234; doi:10.1038/s41467-023-42204-7)
Supplement: Supplementary file 1 — Supplementary Information [file 41467_2023_42204_MOESM1_ESM.pdf]

Supplementary Information for "A super liquid-repellent hierarchical porous membrane for enhanced membrane distillation"

Youmin Hou[1,2], Prexa Shah[1], Vassilis Constantoudis[3], Evangelos Gogolides[3], Michael Kappl[1,*] and Hans-Jürgen Butt[1]

[1]Max Planck Institute for Polymer Research, Ackermannweg 10, 55128, Mainz, Germany
[2]School of Power and Mechanical Engineering, Wuhan University, 430072, Wuhan, China
[3]Institute of Nanoscience and Nanotechnology NCSR Demokritos, 15341 Agia Paraskevi, Greece

[*]Correspondence: kappl@mpip-mainz.mpg.de

Supplementary Note 1. Surface morphology of commercial MD membranes and nanofilament coating

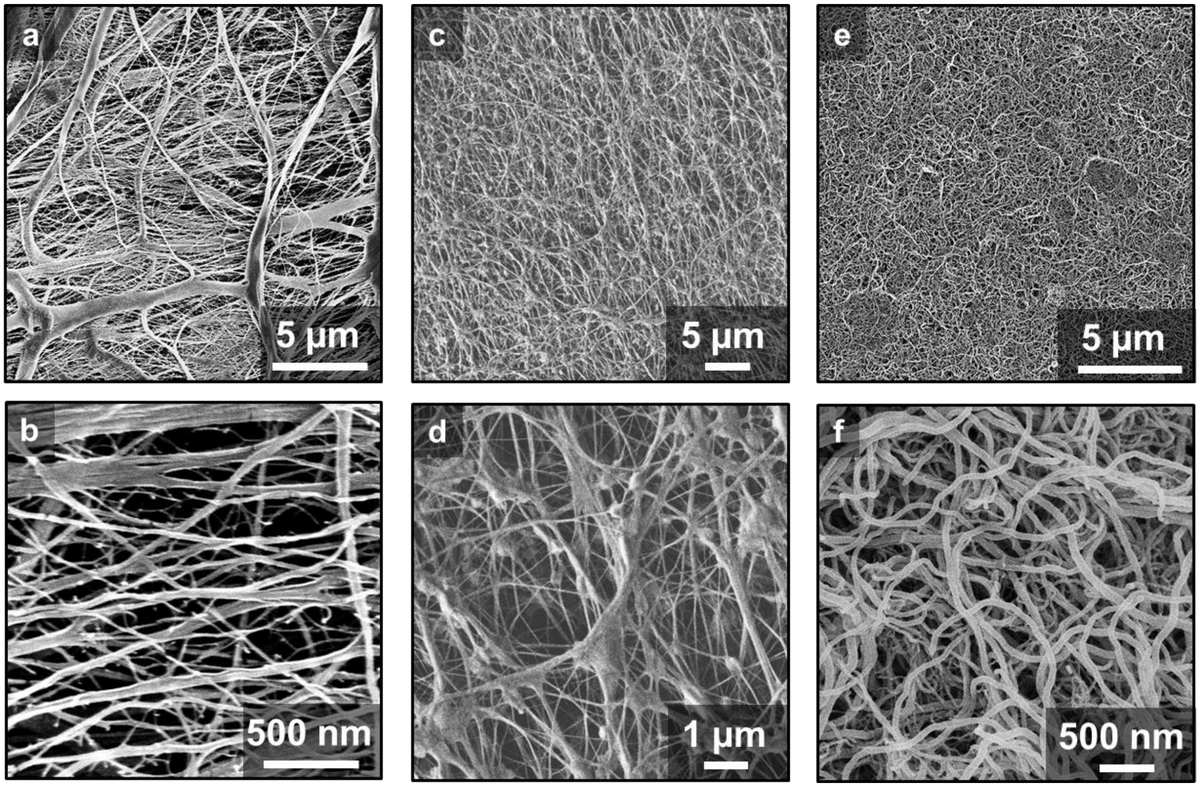

Supplementary Figure 1. Scanning electron microscopy (SEM) images of pristine PE-0.2 (a, b), pristine PTFE-0.2 (c, d), and nanofilament coated PES-0.1 membranes (e, f) with different magnifications.

Supplementary Note 2. Nanofilament coating on PES membranes with different pore sizes

The nanofilaments were coated on commercial PES membranes with different nominal pore diameters by using same fabrication protocol. Although the surface chemistry may slightly differ for PES membranes with different pore sizes owing to possible different manufacturing process, there was barely any difference in morphology among the coatings on the various PES membranes, as shown in Supplementary Figure 2.

The cross-section image of nanofilament-coated PES membranes (Figure S3) demonstrates that the hydrophobic nanofilaments can fully cover the inner structures of membrane, providing a complete hydrophobic non-polar functionalization of the hydrophilic substrate. Considering the surface coverage, the nanofilament coating apparently outperforms the self-assembled monolayer fluoro-silane coating (e.g., perfluorodecyltrichlorosilane) which is commonly used for hydrophobization of surfaces. This is mainly because the coating density of fluoro-silane highly depends on the population of hydroxyl groups on surface and their accessibility for chemical bonding[1]. Practically, not all of the hydroxyl groups on surface react with fluoro-silane, and therefore the monolayer fluorinated coating would inevitably leave some polar sites which decreases the surface hydrophobicity. In contrast, the growth of nanofilaments on surface results in a much higher coverage and better liquid-repellency by the combination of low surface energy and nanotexture.

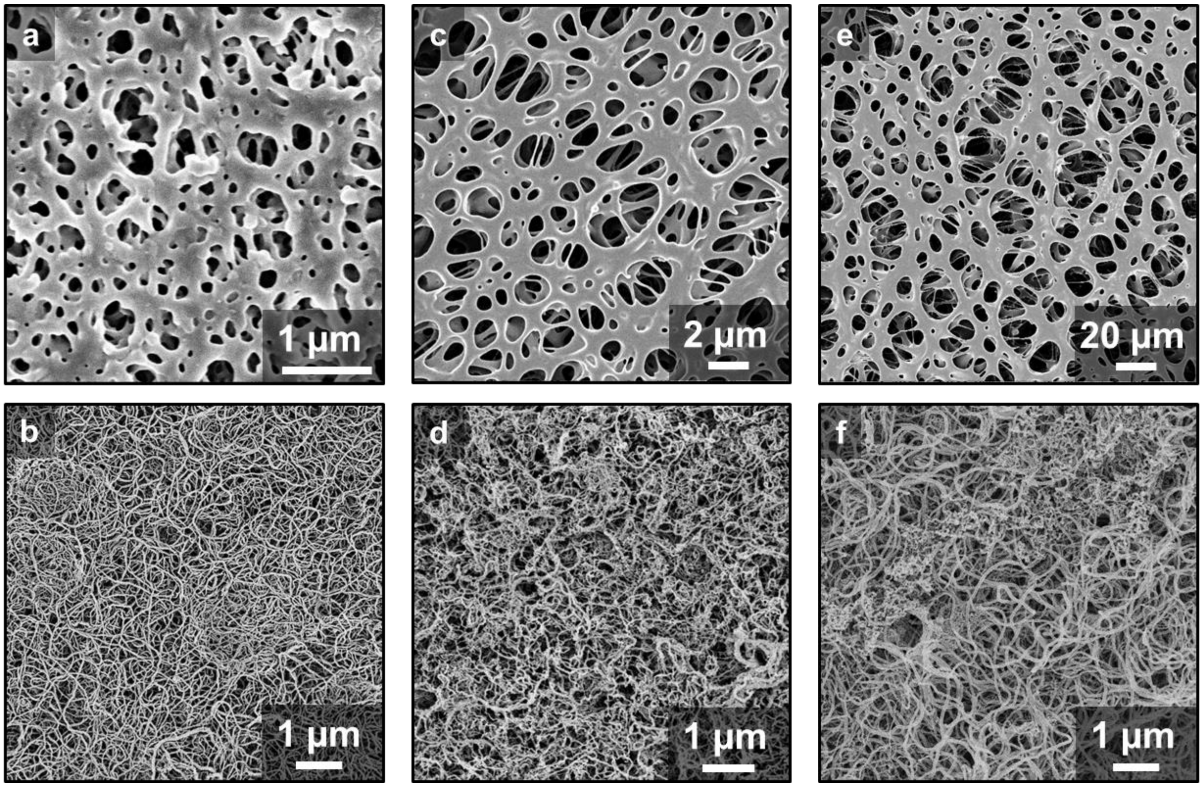

Supplementary Figure 2. Scanning electron microscopy (SEM) images of pristine (a) and nanofilament coated PES-0.1 membrane (b), pristine (c) and nanofilament coated PES-1.2 membrane (d), pristine (e) and nanofilament coated PES-5 membrane (f).

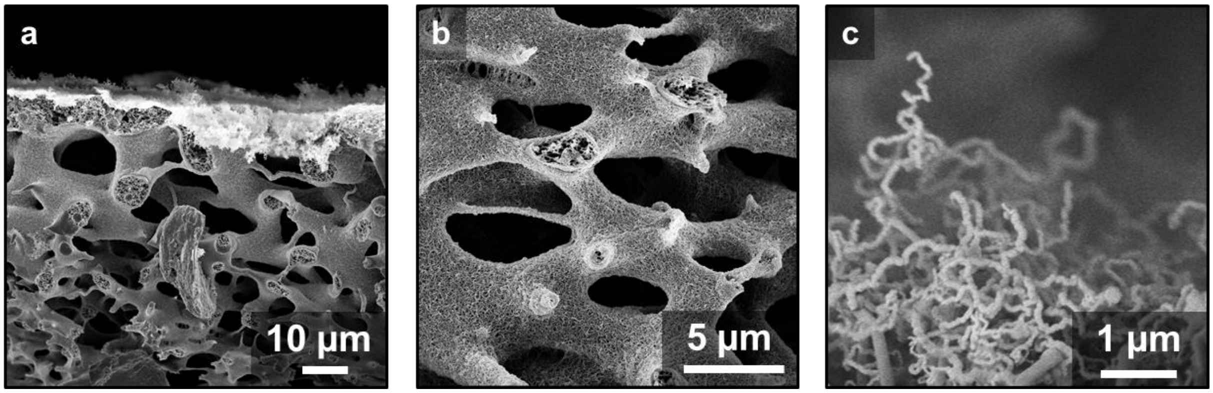

Supplementary Figure 3. Scanning electron microscopy (SEM) images showing the cross-section of nanofilament-coated PES-8 membrane (a) and the coating morphology (b) on the inner surface. The nanofilaments form a nanoscopic overhanging structure (c) on the surface.

Supplementary Note 3. Image analysis of pore size distribution

The size of membrane pores was measured by analyzing the top-down SEM images. The adopted methodology consists of the following steps. First, the image was denoised by means of a standard median filter to remove the salt-pepper image noise. Then, we calculated the intensity histogram and defined the threshold for identifying the membrane pores shape. Next, the image was converted to binary. Morphological operations were applied on the images to get more reliable detection of pores. We finally calculated the positions, sizes, and shape parameters of detected membrane pores. All image processing steps were carried out using self-written software routines.

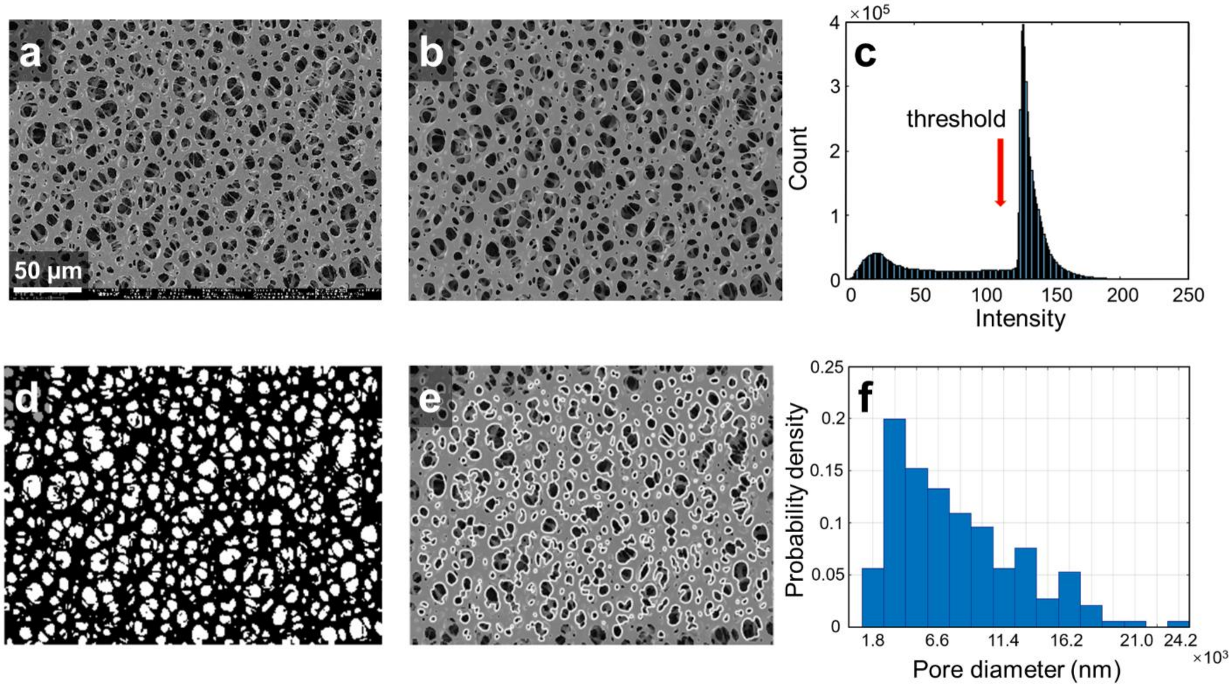

Supplementary Figure 4. Calculation of pore size distribution from SEM image for PES-8 membrane. (a) Initial SEM image. (b) denoised SEM image. (c) Histogram of image pixel intensities. Red arrow denotes the threshold for image binarization. (d) Binary image after the conversion of SEM image. (e) Image showing the detected pores. (f) Statistics of size distribution for the detected pores.

Supplementary Note 4. AFM measurements of nanofilament coated membranes

Surface roughness and morphology of nanofilament-coated PES membranes were analyzed qualitatively by using a Nanowizard IV AFM (JPK Instruments, Berlin, Germany). Standard silicon AFM tips with a nominal tip radius of less than 10 nm (Olympus OMCL-AC240TS) were used for imaging the membrane surface with tapping mode. In a scanning area of 6 x 6 μm^2, the average surface roughness (Ra) and mean square surface roughness (Rq) of commercial PE-0.2 membrane were measured to be 106 nm and 128 nm, respectively (Supplementary Figure 5a). As a comparison, the nanofilament-coated PES-8 membrane exhibited a much higher surface roughness with Ra of 246 nm and Rq of 348 nm, respectively (Supplementary Figure 5b). Though the information extracted from AFM measurements only represented a partial view of top surface topography, the results suggested that the nanofilament-coating increases the surface roughness when compared to the commercial micro-porous membranes.

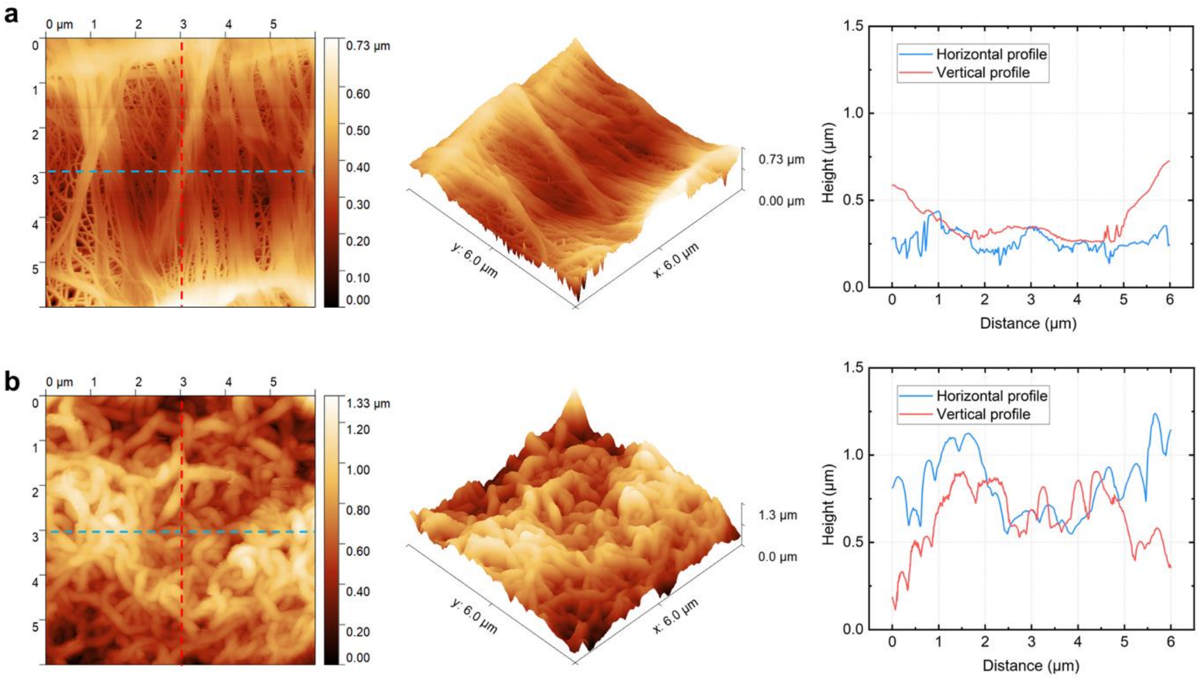

Supplementary Figure 5. 2D, 3D AFM images and typical cross-sections profiles of the PE-0.2 membrane (a) and NF-PES-8 membrane (b). The horizontal and vertical profiles are extracted along the blue dash and red dash lines in AFM images, respectively.

Supplementary Note 5. Setup for liquid entry pressure measurements

The experimental setup used for liquid entry pressure (LEP) measurements consists of a syringe pump, a membrane holder, and a pressure transducer (Supplementary Figure 6a-b)[2,3]. During the measurement, the membrane is mounted inside the membrane holder which connects to the syringe pump and pressure transducer. When salty water in syringe is pumped into the membrane holder with a very low and constant flow rate (0.1 $mLmin^{-1}$), the hydrostatic pressure applied on the tested membrane gradually increases. The variation of pressure in the membrane holder is continuously monitored using a pressure sensor and the data is recorded using a data acquisition system. Once the applied pressure exceeds the capillary pressure of the membrane pores, liquid penetrates the membranes, leading to a pressure drop (Supplementary Figure 6c). The obtained peak value in pressure measurements is regarded as the LEP of tested membrane.

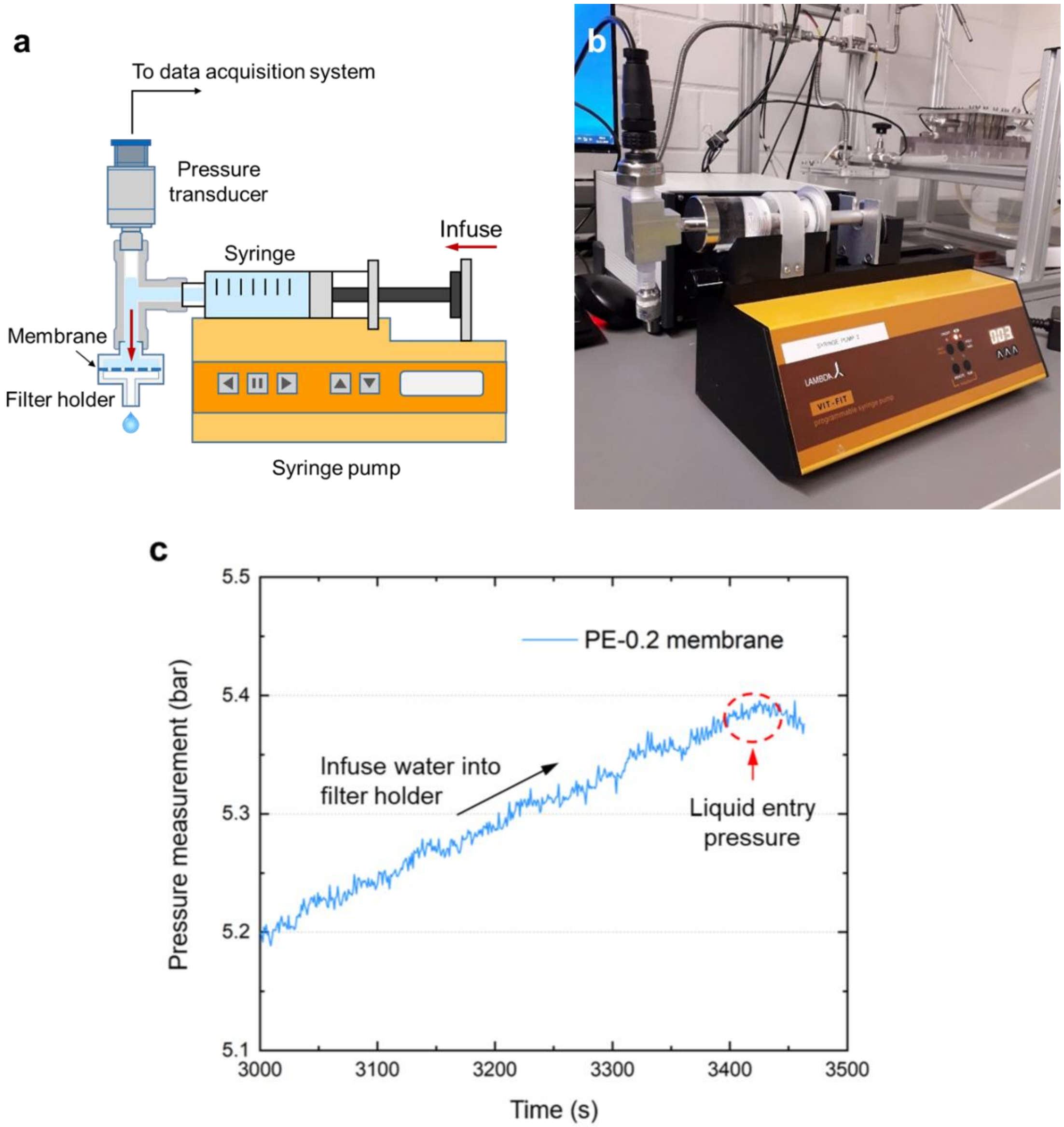

Supplementary Figure 6. Liquid entry pressure (LEP) test (a) Schematic of LEP testing setup (not to scale). (b) Image of LEP testing setup. (c) Pressure as a function of time during a typical LEP test. The pressure drop after the maximum (denoted by red dash circle) is due to membrane wetting by liquid penetration.

Supplementary Note 6. Setup for gas permeability measurements

The experimental setup used for gas permeability measurements consists of a needle regulating valve, a membrane holder, a manometer and a flow sensor (Supplementary Figure 7) [4]. During the measurement, the tested membrane was mounted in the membrane holder which connected to the nitrogen source. When the nitrogen flow passes through the tested membrane, the transmembrane gas pressure was obtained by the manometer and the permeation flux of gas flow was measured by the precise flow sensor (PFMV5, SMC). In this study, we characterized the gas permeability of tested membranes with effective area of 63 mm^2 under different transmembrane pressures.

Supplementary Figure 8 shows the measured gas permeability of commercial PTFE-0.1 and PTFE-0.2 membranes, PES-3 and PES-5 membranes before and after coating with nanofilaments.

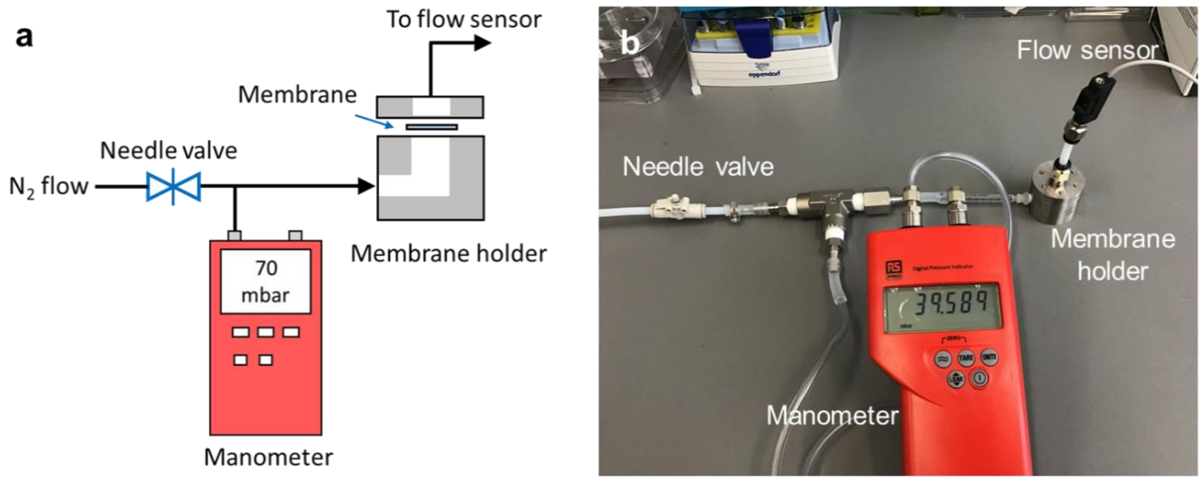

Supplementary Figure 7. Setup for gas permeability tests. (a) Schematic (not to scale). (b) Image of testing setup.

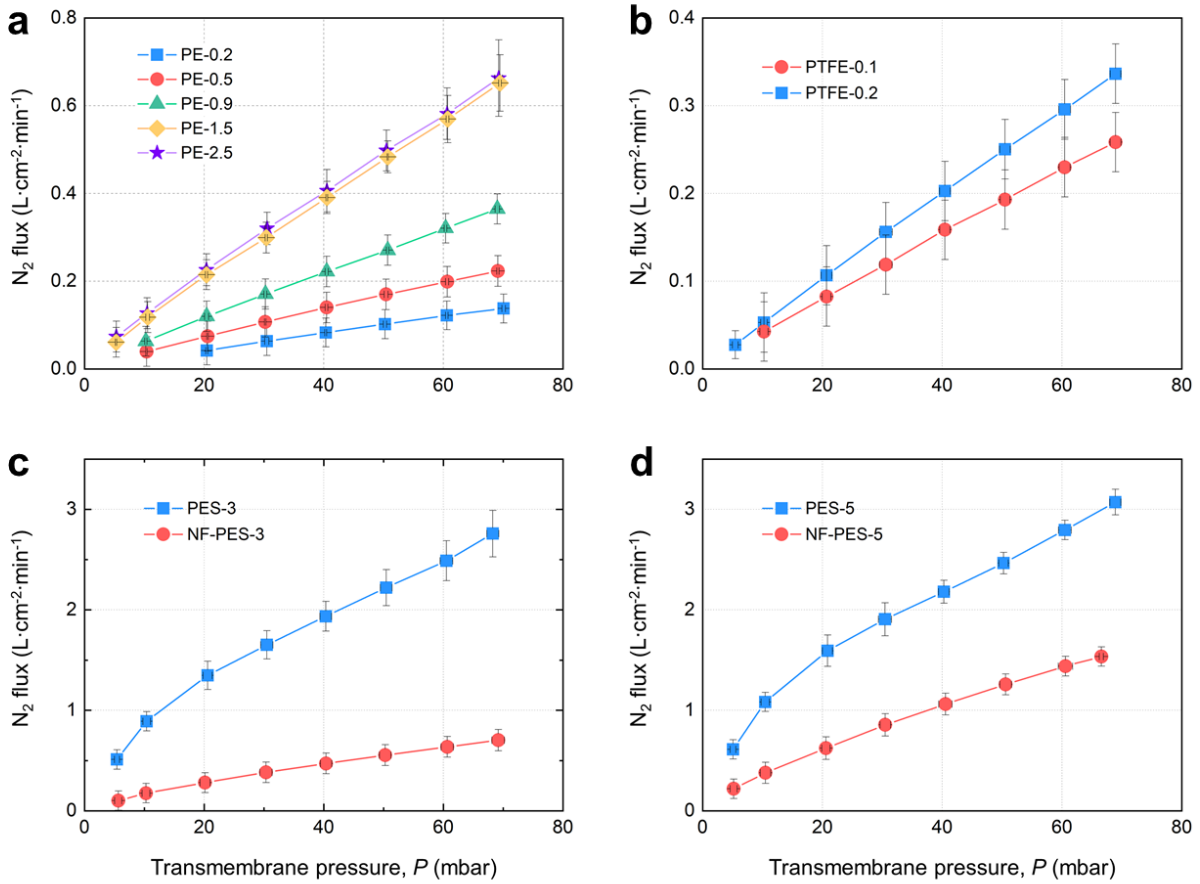

Supplementary Figure 8. Gas permeability tests. Gas flux in dependence of applied pressure for (a) PE membranes, (b) PTFE membranes, (c) PES-3 membranes before and after nanofilament coating, and (d) PES-5 membranes before and after nanofilament coating. Error bars show standard deviation (n=5).

Supplementary Note 7. Test setup for air gap membrane distillation (AGMD)

The lab-scale AGMD testing setup used for this work consists of an AGMD module, feed water and coolant circulating loops, digital balance, conductivity meter, and data acquisition system, as shown in Supplementary Figure 9 [5]. The AGMD module is manufactured from polycarbonate with low thermal conductivity (~0.19W m^{-1} K^{-1}) to reduce the heat loss by conduction. The tested membrane is mounted between a feed flow channel and a condensing surface. A support mesh (~0.5 mm thick) is used to hold the membrane in a proper shape and reduce the membrane deformation due to the pressure difference between feed flow and air gap. An acrylic spacer is used in the MD module to create the required air gap. The total air gap width between membrane and condensing surface is ~4.5 mm.

Feed saline water is heated to the desired temperature and pumped to the AGMD module using magnetic coupling water pump. The hot feed water passes over the membrane surface and returns to the feed container. When water vapor transports through the membrane to the permeate side, it condenses on the copper surface. The condensing surface temperature is controlled by the coolant flow loop using a refrigerated water bath circulator (F25-HE, Julabo). When distilled water slides off the condensing surface by gravity, it is collected in a glass flask. A digital balance (SPX 2202, Ohaus) continuously records the weight of collected distilled water for determining the distillation flux of tested membranes. The conductivities of feed and distilled water are measured by the conductivity meter for calculating the salt rejection during membrane distillation.

For analyzing heat transfer rate and thermal efficiency during distillation process, four Pt100 temperature probes (PM-1/10-1/8-6-0-P-3, Omega) are mounted at the inlet and outlet of feed flow channel and coolant flow channel, respectively. Two flow meters (FT110, Gems) and two pressure transducers (175-5028, RS-Pro) are installed in the pipelines to continuously monitor the flow rate and pressure in the feed and coolant loops. All the sensors in the AGMD testing setup are electrically connected to a data acquisition system, which consists of two National Instrument (NI) analog input modules (PCI 6251 and NI-9216). The measured data during MD experiments are transferred to the computer, which can be monitored in real-time and stored using a self-written LabView code.

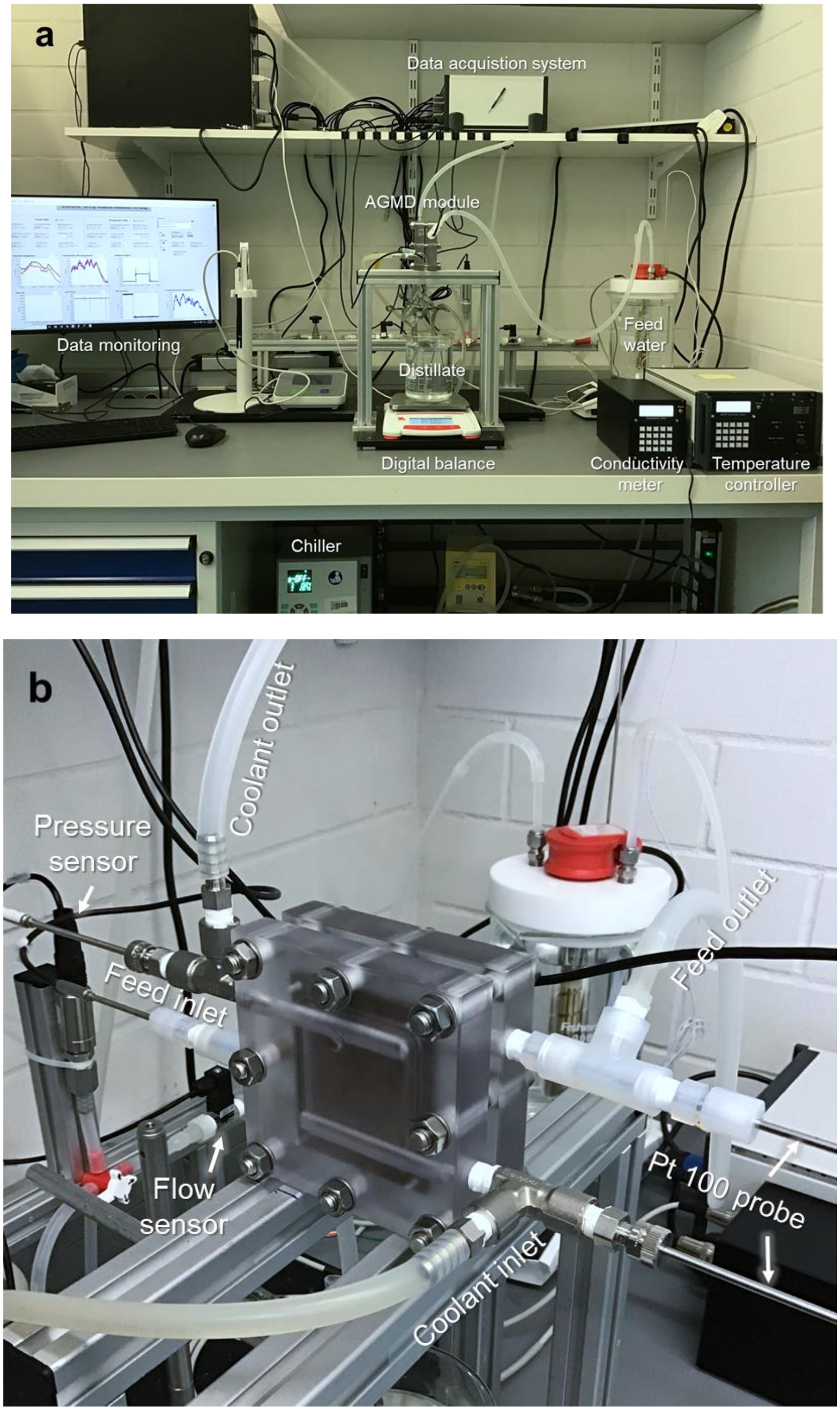

Supplementary Figure 9. Setup for air gap membrane distillation (a) Complete testing setup (b) Image of AGMD module.

Supplementary Note 8. Thermal efficiency of AGMD process

The heat transfer process in air gap membrane distillation (AGMD) can be described by the following three steps. Heat first transfers through the feed side, then across the membrane to the condensing surface, and finally is removed by the coolant flow. In this study, the total heat transfer rate across membrane (q_f) can be estimated by the temperature decrease of feed flow through the feed channel, which is given by [6],

$$q_f = c_f \dot{m}_f (T_i - T_o) = c_f V_f \rho_f (T_i - T_o)$$

in which, c_f is the specific heat capacity rate of feed water, the mass flow rate $\dot{m}_f = V_f \rho_f$ is obtained by the volumetric flow rate $V_f$and density ρ_f, $T_o$and T_i are the feed water temperature at inlet and outlet of feed channel, respectively.

As the thermophysical properties of feed water change with the salinity (S = 54 g kg^{-1}) and water temperature (t_f=50 ~ 80 °C), an empirical correlation with accuracy of 0.1% is used to determine the density (ρ_f in kg m^{-3}) of feed water in AGMD process [7],

$$\rho_f = 10^3 (A_1 F_1 + A_2 F_2 + A_3 F_3 + A_4 F_4)$$

where,

$G_1=0.5, G_2=(2S-150)/150, G_3=2G_2^2-1$

$A_1=4.032G_1+0.115G_2+3.26\times10^{-4}G_3$

$A_2=-0.108G_1+1.571\times10^{-3}G_2-4.23\times10^{-4}G_3$

$A_3=-0.012G_1+1.74\times10^{-3}G_2-9\times10^{-6}G_3$

$A_4=6.92\times10^{-4}G_1-8.7\times10^{-5}G_2-5.3\times10^{-5}G_3$

$A=(2t_f-200)/160, F_1=0.5, F_2=A, F_3=2A^2-1, F_4=4A^3-3A.$

Similarly, the specific heat capacity of feed water (c_f in kJ·kg^{-1}·K^{-1}) is determined by,

$$c_f = A + BT_f + CT_f^2 + DT_f^3$$

where,

$A=5.328-9.76\times10^{-2}S+4.04\times10^{-4}S^2$

$B=-6.913\times10^{-3}+7.351\times10^{-4}S-3.15\times10^{-6}S^2$

$C=9.6\times10^{-6}-1.927\times10^{-6}S+8.23\times10^{-9}S^2$

$D=2.5\times10^{-9}+1.666\times10^{-9}S-7.125\times10^{-12}S^2$

$T_f=t_f+273.15$

In AGMD process, the heat consumed for saline vaporization (q_d) equals to the released heat for water condensation, and therefore q_d can be obtained by the latent heat of distilled water,

$$q_d = \dot{m}_d \, \Delta H_v$$

where $\dot{m}_d$ is the water distillation rate, ΔH_v is the latent heat of water vaporization.

The thermal efficiency of AGMD process, defined as the ratio of the heat transfer rate associated with water distillation over the total heat transfer rate at the feed side, is expressed as

$$\eta_T(\%) = \frac{q_d}{q_f} = \frac{\dot{m}_d \, \Delta H_m}{c_f \, \dot{m}_f \, (T_i - T_o)} \times 100\%$$

Supplementary Table 1 summarizes the uncertainties of the measured parameters including the temperature T, the mass flow rate of feed water G, the.

Supplementary Table 1. Experiment uncertainties of measured parameters

Parameters	Uncertainty (±)
Temperature T (K)	0.07
Volumetric flow rate V_f ($m^3 \cdot s^{-1}$)	3 %
Density of feed water ($kg \cdot m^{-3}$)	0.1 %
Specific heat capacity ($W \cdot m^{-1} \cdot K^{-1}$)	0.28 %
Distillation rate $\dot{m}_d$ ($kg \cdot s^{-1}$)	0.1%

Based on the error propagation, the system uncertainty of heat transfer rate $\sigma(q_f)$ in feed channel is determined by

$$\sigma(q_f) = (T_i - T_o)\left[V_f \, \rho_f \, \sigma(c_f) + c_f \, \rho_f \, \sigma(V_f) + c_f \, V_f \, \sigma(\rho_f)\right] + c_f \, V_f \, \rho_f \, [\sigma(T_i) + \sigma(T_o)]$$

As the error of ΔH_m for the distilled pure water is negligible, the system uncertainty of heat transfer rate $\sigma(q_d)$ is determined by

$$\sigma(q_d) = \Delta H_m \sigma(\dot{m}_d)$$

Therefore, the system uncertainty of thermal efficiency $\sigma(q_d)$ in AGMD process is given by

$$\sigma(\eta_T) = \sqrt{\left[\frac{\sigma(q_d)}{q_f}\right]^2 + \left[\frac{q_d}{q_f^2}\sigma(q_f)\right]^2}$$

Supplementary Note 9. Membrane fouling test

To test the fouling resistance of the NF-PES membrane, we immersed the membrane in a NaCl and Bovine serum albumin (BSA) solution (concentration of BSA is 500 mgL^{-1}). The BSA is used as a model organic foulant to assess the anti-fouling performance of membranes. Supplementary Figure 10 shows the variation of droplet contact angle on PE, PTFE, and NF-PES membranes after the immersion test in the NaCl-BSA mixed solution. The droplet contact angle on the PE and PTFE membranes decreases sharply after being immersed in the NaCl-BSA solution, indicating the membrane fouling due to the BSA adhesion. By contrast, the NF-PES membrane maintains the superhydrophobicity after a 24-hour immersion test. The stable droplet contact angle confirmed the high resistance of fouling owing to the coated nanofilaments.

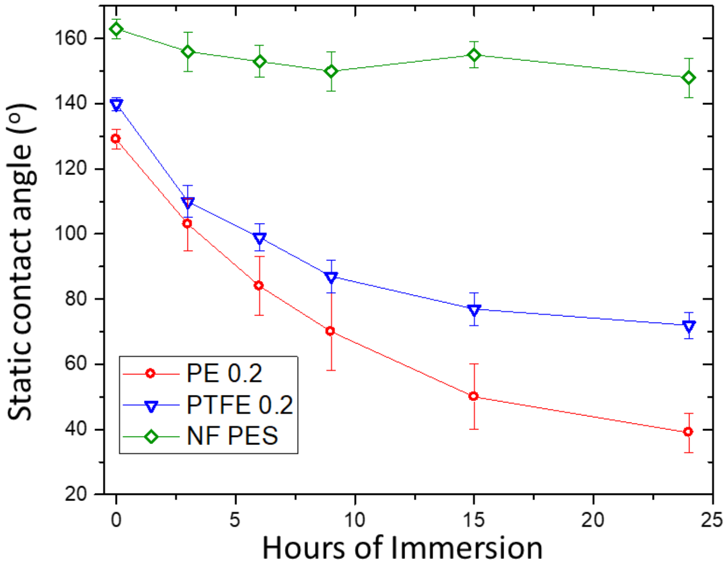

Supplementary Figure 10. Membrane fouling test. Decrease of wetting resistance over time characterized by contact angle of the water droplet on the PE-0.2, PTFE-0.2, and nanofilament-coated PES membranes as a function of immersion time in NaCl-BSA solution. The concentration of BSA is 500 mg L^{-1}. Error bars show standard deviation (n=5).

Supplementary Note 10. Change in Wetting properties of bulk PTFE sample after immersion in hot distilled water (80°C)

A commercial PTFE foil (1 mm thickness) was immersed into hot distilled water for 24 hours. Advancing and receding contact angle of distilled water on this PTFE film was measured before and after immersion. A clear reduction of water repellency was observed as both contact advancing and receding contact angles were reduced and contact angle hysteresis was increased (Supplementary Figure 11).

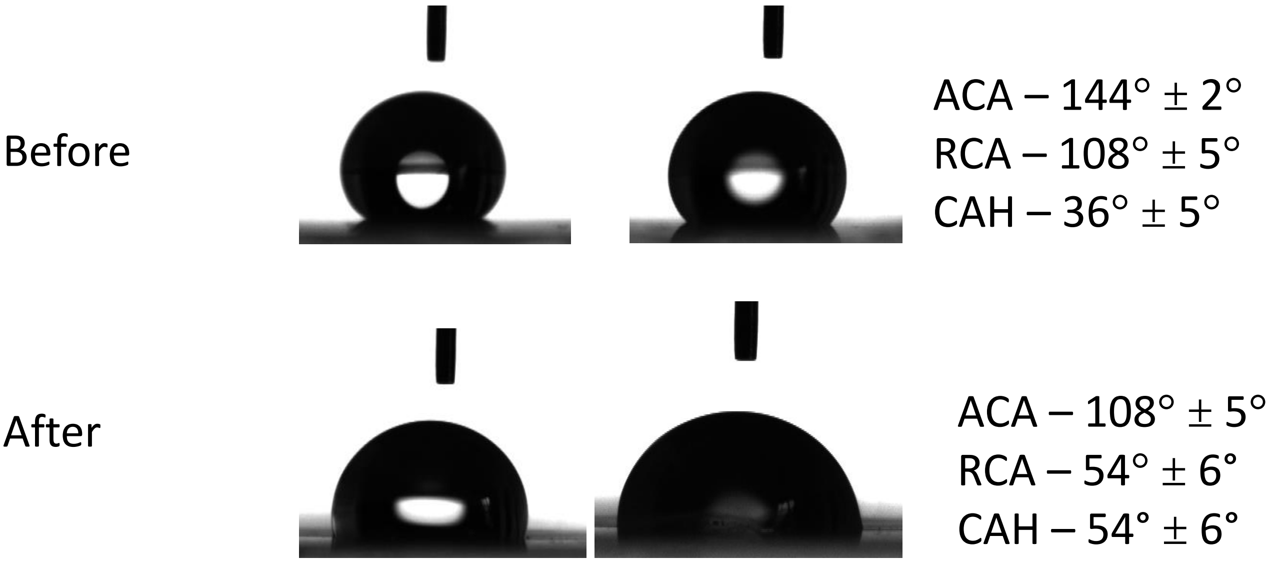

Supplementary Figure 11. The wetting properties of a bulk PTFE sample before and after immersion for 24 hours in hot distilled water (80°C). The immersion leads to decreasing of advancing (ACA) and receding (RCA) contact angles as well as to increase in contact angle hysteresis (CAH), indicating reduced hydrophobicity after immersion test.

Supplementary References

1. Bhushan B, Hansford D, Lee KK. Surface modification of silicon and polydimethylsiloxane surfaces with vapor-phase-deposited ultrathin fluorosilane films for biomedical nanodevices. *J. Vac. Sci. Technol., A* **24**, 1197-1202 (2006).

2. Guo F, Servi A, Liu A, Gleason KK, Rutledge GC. Desalination by Membrane Distillation using Electrospun Polyamide Fiber Membranes with Surface Fluorination by Chemical Vapor Deposition. *ACS Applied Materials & Interfaces* **7**, 8225-8232 (2015).

3. Alkhudhiri A, Darwish N, Hilal N. Membrane distillation: A comprehensive review. *Desalination* **287**, 2-18 (2012).

4. Khayet M, Matsuura T. Preparation and Characterization of Polyvinylidene Fluoride Membranes for Membrane Distillation. *Industrial & Engineering Chemistry Research* **40**, 5710-5718 (2001).

5. Khalifa A, Lawal D, Antar M, Khayet M. Experimental and theoretical investigation on water desalination using air gap membrane distillation. *Desalination* **376**, 94-108 (2015).

6. Shahu VT, Thombre SB. Air gap membrane distillation: A review. *Journal of Renewable and Sustainable Energy* **11**, 045901 (2019).

7. Sharqawy MH, Lienhard JH, Zubair SM. Thermophysical properties of seawater: a review of existing correlations and data. *Desalination and Water Treatment* **16**, 354-380 (2010).
